# The proliferation and angiogenesis in hemangioma-derived endothelial cells is affected by STC2 medicated VEGFR2/Akt/eNOS pathway

**DOI:** 10.12669/pjms.39.4.7384

**Published:** 2023

**Authors:** Shanshan Ren, Yuchang Yang

**Affiliations:** 1 Shanshan Ren, Department of Plastic Surgery, The Affiliated Hospital of Weifang Medical University, Weifang, Shandong, 261000, China; 2 Yuchang Yang, Department of Plastic Surgery, The Affiliated Hospital of Weifang Medical University, Weifang, Shandong, 261000, China

**Keywords:** Hemangioma, Angiogenesis, Stanniocalcin2, Tube formation

## Abstract

**Objective:**

Stanniocalcin-2 (STC2), a secreted glycoprotein that is involved in the regulation of angiogenesis, was proposed as one of the mechanisms of neovascularization in hemangioma (HA). We aimed to investigate the effect of STC2 on proliferation and angiogenesis in hemangioma-derived endothelial cells.

**Methods:**

The hemangioma samples from HA patients with the median age of six months were surgically collected in the Affiliated Hospital of Weifang Medical University from October 2019 to June 2021, and divided into normal skin tissues (n=10), involuting-phase HAs (n=10) and proliferating-phase HAs (n=10) according to the Mulliken classification. The expression of STC2 was detected in involuting-phase HAs and proliferating-phase HAs. Hemangioma endothelial cells (HemEC) were transfected with small interfering RNA (siRNA) specific for STC2, and cell survival and tube formation were analyzed.

**Results:**

STC2 expression in proliferating-phase HAs was markedly higher than in the normal skin tissues and involving-phase HAs. Similarly, STC2 expression was higher in HemEC compared to the control human umbilical vein endothelial cells (HUVEC). Knockdown of STC2 slowed the proliferation of HemEC and decreased the expression of proliferating cell nuclear antigen (PCNA) in HemEC. Moreover, knockdown of STC2 in HemEC inhibited vascular endothelial cell angiogenesis and regulated the expression and phosphorylation of vascular endothelial growth factor receptor 2 (VEGFR2). Mechanistically, STC2 knockdown attenuated the activation of Akt/eNOS signaling, which was abolished by insulin growth factor-1 (IGF-1), the activator of Akt signaling, accompanying by increased proliferation and tube formation of HemEC.

**Conclusion:**

Inhibition of STC2 suppresses HemEC proliferation and angiogenesis by VEGFR2/Akt/eNOS pathway, which warrants further development of STC2-based strategies for HA treatment.

## INTRODUCTION

Hemangioma (HA) is one of the most common benign tumors,^1^ formed by abnormal proliferating vascular endothelial cells. It occurs in about 5% to 10% of infants,^2^ affects physical characteristics, causing pain, deformation and eating difficulties, and in severe cases may be life-threatening.^3^ Understanding the underlying mechanisms of HA are important for the successful treatment and prevention of this condition. Angiogenesis or formation of new blood vessels in the pre-existing vascular system, is a decisive event leading to tumor growth and metastasis.^4^ Studies show that the activation of the vascular endothelial growth factor receptor-2 (VEGFR2),^5^ a crucial target of angiogenesis, initiates the recruitment and migration of endothelial cells to the tumor.^6^ Therefore, in certain cancers, treatment with VEGFR2 inhibitors can have anti-angiogenic effect.^7^

Stanniocalcin-2 (STC2) encodes a secreted glycoprotein that is involved in many physiological processes.^8^ Increasing evidence show dysregulated STC2 in solid tumors.^9^ Studies showed that STC2 mediated VEGF-C/VEGF-D/VEGFR-3 pathway in colorectal cancer to regulate epithelial–mesenchymal transition (EMT) -related molecules through increasing VEGFR *in vitro*, and accelerated angiogenesis of human umbilical vascular endothelial cells (HUVECs) through the VEGF/VEGFR2 signaling pathway.^10^ However, the link between STC2 expression and hemangioma remains unclear. In this study, an infantile primary hemangioma endothelial cells (HemEC) were used to explore the potential function of STC2 in HA.

## METHODS

The hemangioma samples from HA patients with the median age of six months (female/ males: 16/4) were surgically collected in the Affiliated Hospital of Weifang Medical University from October 2019 to June 2021, and divided into normal skin tissues (n=10), involuting-phase HAs (n=10) and proliferating-phase HAs (n=10) according to the Mulliken classification.^11^ Informed consent was obtained from the guardians of all HA patients and the protocol of the study was approved by the Ethics Committee of the Affiliated Hospital of Weifang Medical University (Approval No.: Wyfy-2021-ky-165; Date: December 12, 2021).

The human umbilical vein endothelial cells (HUVECs, Cell Bank of the Chinese Academy of Sciences, Shanghai, China) and mouse hemangioendothelioma cell EOMA (CRL-2586, ATCC, Rockville, MD, USA) were maintained in M200 medium (Sigma-Aldrich, St. Louis, MO, USA) and RPMI1640 (Sigma-Aldrich) medium, respectively, supplemented with penicillin/streptomycin (Sigma-Aldrich). In addition, hemangioma endothelial cells (HemEC) isolated from proliferating-phase HA of three infants were cultured in Endothelial Basal Medium-2 (EBM-2) (Sigma-Aldrich). All mediums contained 10% fetal bovine serum (FBS; Gibco BRL, Grand Island, NY, USA) and cultured at 37ºC in a humid CO_2_ incubator.

Total RNA was extracted from tissues or cells, and 2ng of a total RNA was used for cDNA synthesis using five All-In-One RT MasterMix (Takara, Biotechnology Co., Ltd., Dalian, China). The reverse transcription quantitative polymerase chain reaction (RT-QPCR) was performed using a thermocycler (Bio-Rad, California, USA). Cycling conditions were as follows: initial denaturation step of 95 °C for 10 minutes followed by 35 cycles of 95 °C denaturation for 15 seconds and an annealing/extension step of 30s at 60 °C. The primers of STC2 were purchased from Shanghai Generay Biotech Co., Ltd.

### The following primer sequence was used:

STC2 forward: 5’-GGGTGTGGCGTGTTTGAATG-3’

STC2 reverse 5’-TTTCCAGCGTTGTGCAGAAAA-3’

GAPDH forward 5’-GGAGCGAGATCCCTCCAAAAT-3’

GAPDH reverse 5’-GGCTGTTGTCATACTTCTCATGG-3’

For the Western blot analysis, cells were lysed in ice-cold RIPA lysis buffer (Beyotime Institute of Biotechnology), and the protein content was determined using a Bradford assay kit (Sigma-Aldrich). GAPDH was used as a loading control. Each protein sample (8 μg) was separated on 10% SDS-PAGE, transferred onto a PVDF membrane Sigma-Aldrich) and incubated with the specified primary and secondary antibodies: phospho-Akt (Ser 473) (ab81283, 1/5000), Akt (ab8805, 1/500), phospho-eNOS (Ser1177) (ab215717, 1/1000)., eNOS (ab76198, 1/500), STC2 (ab255610, 1/1000), phospho-VEGFR2 (Tyr1175) (ab194806, 1/500), VEGFR2 (ab134191, 1/1000), GAPDH (ab8245, 1/1000), PCNA (ab29, 1/500), Goat Anti-Rabbit IgGs (ab205718 and ab150077, 1/1000; ab97051, 1/2000). Proteins were visualized using ECL (Pierce, Shanghai, China).

Three small interfering RNA (siRNA) sequences, targeting STC2 (si-STC2), and the negative control siRNA (si-NC) with no definite target were purchased form GenePharma Co., Ltd (Shanghai, China). HemEC were plated in 6-well plates 24 hours before siRNAs transfection using the commercial reagent (Lipofectamine 2000, USA). At 48 hours post-transfection, cells were treated with 500 μg/ml neomycin (Sigma) for four weeks for selection.

Twenty-four, 48 and 72 hours after the transfection, cell viability was measures using Cell Counting Kit-8 (Dojindo Molecular Technologies, Inc., Kumamoto, Japan), and 20 *μ*l of the reagent was added to HemEC at 37°C for four hours, followed by the measurement of the absorption of cell solution at 450 nm using multiplate reader (Synergy HT, Winooski, VT, USA).

HemEC were pre-coated with Matrigel (Corning, New York City, USA) and serum-free EBM-2 (1:1). The examination and calculation of tube formation in HemEC were performed using a fluorescence microscope (Axio Observer A1, Carl Zeiss, Baden-Wuerttemberg, Germany) and an imageJ software (NIH, Bethesda, MD, USA).

### Statistical analysis:

The measurement data (mean ± SD) were analyzed by SPSS statistical software (Version 22 for Windows; SPSS, Chicago, IL, USA) by One-way ANOVA and Tukey’s HSD test, using a threshold of P lower than 0.05 as statistically significant.

## RESULTS

**Fig.1:**
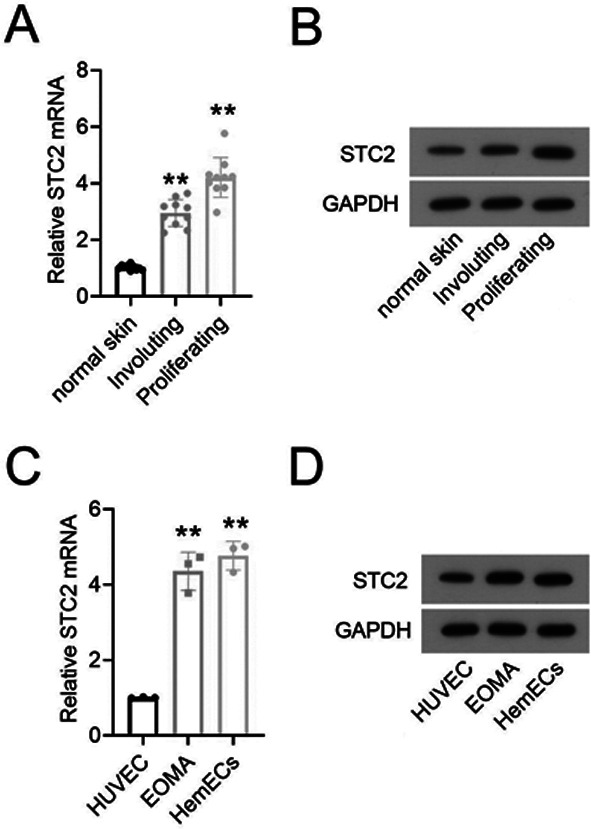
The expression of STC2 was upregulated in HA tissues and cells. The expression of STC2 mRNA (A) and protein (B) in normal skin tissues, involuting-phase HAs and proliferating-phase HAs was analyzed using qRT-PCR and western blot assay (n=10). The expression of STC2 mRNA (C) and protein (D) in HUVEC, EOMA and HemEC was analyzed using qRT-PCR and western blot assay.

As shown in Fig.1A and 1B, there was a significantly higher expression levels of STC2 mRNA and protein in proliferating-phase HAs compared to the normal skin tissues and involving-phase HAs. Similarly, expression of STC2 was increased in EOMA and HemEC as compared with HUVEC (Fig.1C and 1D).

**Fig.2:**
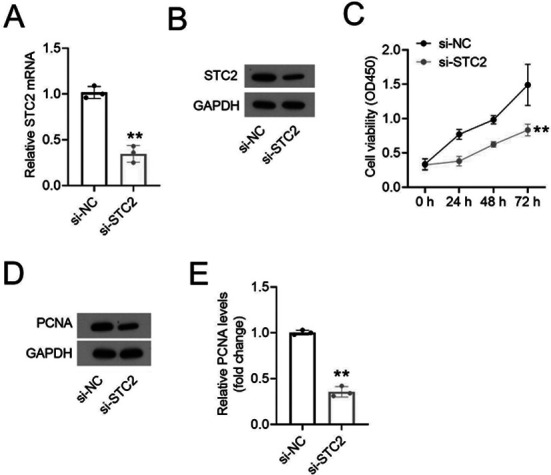
Knockdown of STC2 inhibited HemEC proliferation. The mRNA (A) and protein (B) expression of STC2 in HemEC transfected with si-STC2 or si-NC. (C) HemEC proliferation of was determined using CCK-8 assay. (D-E) PCNA was analyzed via western blot.

Knockdown of STC2 by siRNA successfully reduced STC2 expression in HemEC (Fig.2A and 2B). The proliferation of vascular endothelial cells is a key factor affecting tumor-related angiogenesis.^21^ STC2 inhibition also significantly reduced the HemEC proliferation (Fig.2C) with the reduced protein expression of Proliferating Cell Nuclear Antigen (PCNA), a marker of cell proliferation (Fig.2D and 2E).

**Fig.3:**
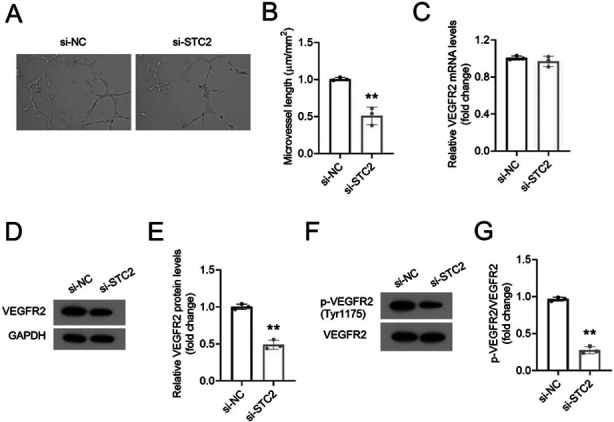
STC2 knockdown inhibited tube formation of HemEC in vitro. Tube formation assay showed si-STC2 suppressed vascular formations of HemEC. Bar = 0.1 mm. (B) Comparison of the total tube length from. (C) qRT-PCR analysis of VEGFR2 in HemEC. (D-G) The expression of VEGFR2 and p- VEGFR2 in HemEC was assessed by western blot.

Compared to si-NC cells, si-STC2 HemEC cells had shortened tube lengths, reduced number of junctions and broken tube networks (Fig.3A and 3B). Levels of VEGFR2 mRNA in si-STC2-transfected HemEC were similar to those of si-NC-treated cells (Fig.3C). However, the expression of VEGFR2 protein in si-STC2-treated cells was significantly down-regulated (Fig.3D and 3E). Additionally, the phosphorylation of VEGFR2 (Tyr1175) in cells treated with si-STC2 was reduced (Fig.3F and 3G).

**Fig.4:**
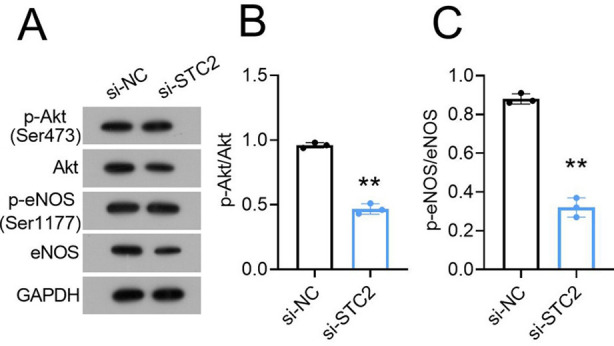
STC2 knockdown surppressed Akt/eNOS signaling of HemEC *in vitro*. (A) Western blot analysis of p-Akt and p-eNOS in HemEC transfected with si-STC2 or si-NC. (B-C) Comparison of p-Akt/Akt and p-eNOS/eNOS.

**Fig.5:**
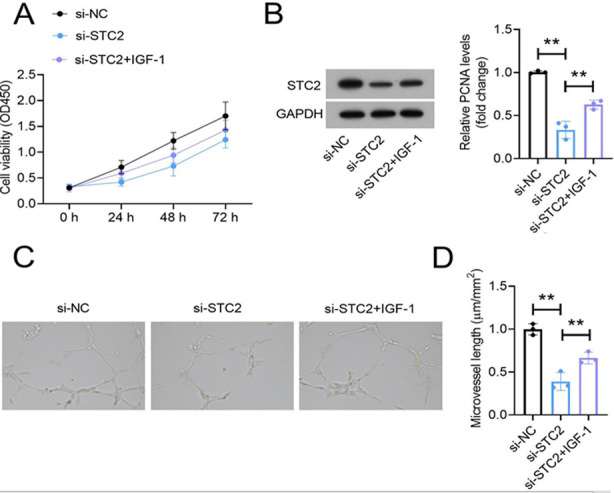
IGF-1 abolished the effects of STC2 siRNA on the proliferation and tube formation of HemEC. The HemEC proliferation were detected using CCK-8 assay. (B) PCNA expression was analyzed using western blot assay. (C-D) Tube formation assay showed si-STC2 suppressed vascular formations of HemEC, which could be reversed by IGF-1. Bar = 0.1 mm.

As shown in Fig.4A-4C, STC2 knockdown significantly reduced the levels of phosphorylated Akt (p-Akt) and eNOS (p-eNOS) in HemEC. Moreover, treating si-STC2-transfected HemEC with 50 ng/ml Insulin-like growth factor one (IGF-1; the activator of Akt pathway) for 48 hours abolished the inhibitory effect of si-STC2 on HemEC proliferation (Fig.5A and 5B) and angiogenesis (Fig.5C and 5D).

## DISCUSSION

To investigate the potential role of STC2 in HA processes, we examined the expression of STC2 in normal skin tissues, involuting-phase HAs and proliferating-phase HAs. The results showed that the expression levels of STC2 mRNA and protein were significantly higher in proliferating-phase HAs compared to normal skin tissues and involuting-phase HAs. Previous studies have showed that the elevated expression of STC2 act as pro-survival factor that contributes to tumor dormancy of breast cancer, increases cancer aggressiveness by increasing angiogenesis and nutrient supply to the tumor and is associated with the poor prognosis of malignant tumors.^12^ However, breast cancer patients with high expression of STC2 have longer overall survival (OS),^13^ suggesting controversial prognostic value of STC2 in solid tumors. Therefore, STC2 may be an oncogene for the occurrence and development of HA. Moreover,, we also found that STC2 was increased in HA cells, and its silencing inhibited proliferation and tube formation of HemEC in vitro, as indicated by the reduced levels of the Proliferating Cell Nuclear Antigen (PCNA), a marker of cell proliferation.^14^ Our results suggest that targeting STC2 might help inhibit the development and progression of HA.

We found that STC2 silencing suppressed the protein expression of VEGFR2. Moreover, STC2 silencing attenuated the phosphorylation of VEGFR2 (Tyr1175) in HemEC. VEGFR2 is essential for angiogenesis, and a small amount of VEGFR2 can maintain angiogenesis to a certain extent.^15^ Previous studies showed that in mice VEGFR2 phosphorylation at site 1173 (corresponding to 1175-Tyr in humans) was essential for endothelial and hematopoietic cells.^16^ Based on these results, we may speculate that STC2 may promote tumor growth and angiogenesis via modulating VEGFR2. Previous studies have shown that the signal network initiated by VEGFA/VEGFR2 is the key to the formation of new blood vessels, thus resulting in abnormal cell proliferation.^17^ For instance, placenta growth factor (PIGF), a member of the VEGF superfamily, stimulates the secretion of VEGFA to enhance the interaction of VEGFA and VEGFR2, thereby promoting angiogenesis.^18^ However, whether STC2 is involved in regulating the secretion of VEGFA to affect VEGFR2 signal transduction still remains unclear.

The angiogenesis and survival of tumor cell induced by VEGFR2 involves several complex signaling pathways,^19^ including PI3K/Akt signaling pathway.^20^ Akt is a key molecule in this signaling pathway, and plays a crucial role in regulating VEGFR2 function.^21^ We showed that STC2 silencing significantly inhibited the Akt and eNOS phosphorylation, and this inhibition was abolished by IGF-1, an activator of Akt/eNOS pathway. Additionally, Akt activates endothelial nitric oxide synthase (eNOS) that could be produced by endothelial cells, participates in the regulation of cell permeability,^22^ reflect the endothelial activity, and maintains endothelial function.^23^ Studies have shown that the acceleration of tumor cell proliferation caused by STC2 possibly occurs via the activation of the PI3K/AKT pathway,^23^ which is consistent with our result. These results indicate that the VEGFR2/Akt/eNOS pathway plays a crucial role in STC2-mediated cell growth and angiogenesis in HA.

### Limitations:

First, we lacked clinicopathological characteristics and follow-up information of clinical samples. Second, further studies are still needed to elucidate the exact mechanism by which STC2 regulates VEGFR2, which will be the subject of a future study.

## CONCLUSION

STC2-mediated activity of VEGFR2/Akt/eNOS promotes HemEC proliferation and angiogenesis. Our results may help in developing new approaches in the treatment of HA.

### Consent for publication:

All authors (SSR, MJL and YCY) agreed the submission and abided by the policy of the journal and copyright.

### Author Contributions:

**SR and ML:** conceived and designed the study.

**YY:** collected the data and performed the analysis.

**SR and ML:** were involved in the writing of the manuscript and is responsible for the integrity of the study.

All authors have read and approved the final manuscript.
